# Supplementary material for: Targeting of the ELR+CXCL/CXCR1/2 Pathway Is a Relevant Strategy for the Treatment of Paediatric Medulloblastomas
Source: Cells. 2022 Dec 5;11(23):3933. doi: 10.3390/cells11233933 (PMC9738107; doi:10.3390/cells11233933)
Supplement: Supplementary file 1 [file cells-11-03933-s001.zip › cells-1994966-Supplementary.pdf]

cells MDPI

Supplemental Materials and methods

Reagents and antibodies

All reagents were purchased from Sigma-Aldrich, unless otherwise stated.

Immunoblotting antibodies

Anti-AKT (AKT, Cell Signalling Technology CST, Danvers, USA, #9272S), anti-phospho-AKT (CST, S473 #9271), anti-ERK (CST, #4695S), anti-phospho-ERK (CST, T202/Y204, #4370S), anti-HSP90 (CST, #4877S). Anti-ARD1 was a homemade polyclonal rabbit antibody, which was previously described [63]. HRP-coupled secondary antibody anti-rabbit IgG was from Promega, Charbonnières-les-Bains, France.

Primers	Nucleotide sequence	Purchased from
CXCR1 F	5' TTAAGTCACTCTGATCTCTGAC	Sigma-Aldrich
CXCR1 R	5' TGGTTTGATCTAACTGAAGC	Sigma-Aldrich
CXCR2 F	5' CCAGTCAGGATTTAAGTTTACC	Sigma-Aldrich
CXCR2 R	5' GTTGATTTCCAGGGATTCTG	Sigma-Aldrich
CXCL1 F	5' ATGCTGAACAGTGACAAATC	Sigma-Aldrich
CXCL1 R	5' TCTTCTGTTCCTATAAGGGC	Sigma-Aldrich
CXCL2 F	5' CTGCTCCTGCTCCTGGTG	Sigma-Aldrich
CXCL2 R	5' AGGGTCTGCAAGCACTGG	Sigma-Aldrich
CXCL3 F	5' CCTCAAGAACATCCAAAGTG	Sigma-Aldrich
CXCL3 R	5' CCCCTTGTTCAGTATCTTTTC	Sigma-Aldrich
CXCL5 F	5'GATGATCAAAAGTCCTGATCCA	Sigma-Aldrich
CXCL5 R	5' CTGCAGCCACTGGTTCTGT	Sigma-Aldrich
CXCL7 F	5' GTACGCGGACTTCAGACAGAT	Eurogentec (Angers, France)
CXCL7 R	5' CGCGACAGACACTGCATAAC	Eurogentec (Angers, France)
CXCL8 F	5' GTTTTTGAAGAGGGCTGAG	Sigma-Aldrich
CXCL8 R	5' TTTGCTTGAAGTTTCACTGG	Sigma-Aldrich
36B4 R	5' GGCCAGGACTCGTTTGTACC	Sigma-Aldrich
36B4 F	5' CAGATTGGCTACCCAACTGTT	Sigma-Aldrich

FACS antibodies (Miltenyi Biotech).

Anti-CXCR1 (CD181) antibodies, anti-human, APC no. 130-105-353 and its isotype Control IgG2b APC no. 130-122-932.

Anti-CXCR2 (CD182) antibodies, anti-human, PE no. 130-100-926 and its isotype Control IgG1 PE no. 130-113-438.

Human qPCR primers.

F=forward; R=reverse.

Cell culture

X-ray resistant cells

Highly confluent Daoy and HD-MB03 cells (two populations each, DR1/DR2 and HR1/HR2 respectively for irradiated resistant Daoy and HD-MB03cells) were irradiated at 8Gy by photon every week for 10 weeks, using a Faxitron cabinet X-ray irradiator (160kV-6·3 mA; Edimex, Le Plessis-Grammoire, France). After 10 weeks, naïve and resistant cells were irradiated once at 8 Gy and cell viability was determined 5 days later using the ADAM cell counter (MBI, Dorval, Canada). Viability was close to 80% for both Daoy and HD-MB03 resistant cells, while naïve Daoy cells survived at 60% after one such irradiation and naïve HD-MB03 at only 40% [55].

All cells were cultured at 37°C, in a 5% CO_2 incubator. For all experiments, cell lines were maintained for no more than 2 months. The absence of mycoplasma was verified

each month by using PlasmoTest Kit (#10L07-MM, InvivoGen, Toulouse, France). Daoy and ONS-76 belong to the Shh genetic subgroup, D341-Med and HD-MB03 to group 3.

Normal cells

Mouse cerebellar astrocytes C8D1A (CRL -2541) were purchased from the American Tissue Culture Collection ATCC. These cells were cultured in DMEM (1X) + GlutaMAX (Invitrogen) medium supplemented with 10% FBS (D. Dutscher). Cells were cultured at 37°C in a 5% CO_2 incubator.

Cell proliferation

1500 Daoy, ONS -76, HD -MB03 or D341 Med cells were sown in triplicate in plates with six wells and the cells were counted daily for 8 days using a Coulter counter (Villepinte). The relative number of cells (compared to day 1) was determined daily. Cell growth was fitted to an exponential growth equation:

$Y = Y0 * \exp(k * X)$ (Y= Cell number at day X; Y0= Cell number at day 0 (graphically calculated); X= Day following cell plating (cells plated at X0=0); k= Exponential growth constant rate. Doubling times were compared.

Clonogenicity assays

150 Daoy/DR1/DR2/ ONS -76 and 300 HD -MB03/HR1/HR2 cells were seeded in triplicate in 6-well plates in the presence or absence of treatment (1 or 2·5μM MCK140 or DMSO as control). On day 9 (Daoy/ONS-76) and day 11 (HD -MB03), cells were fixed in ice-cold absolute ethanol (Sigma) for 20 min, washed with PBS and then stained with Giemsa 50% for 30 min. The number of clones was quantified using ImageJ software. We only considered clones containing more than 50 cells.

Cell death assays

75000 Daoy/ ONS-76/DR1/DR2/C8D2A and 150000 HD-MB03/D341Med/HR1/HR2 were seeded in 6-well plates. After 2 days, cells were treated with control (DMSO) or C29 (1, 2·5, 5μM) for 48 hours. After 48 hours, the medium was recovered, and the adherent cells were dissociated and added to the supernatant. Cell death was detected by PE channel using FACS Cytoflex (Beckman) immediately after labelling the cells with 1ug/ml propidium iodide (Miltenyi Biotec). Data were presented with the percentage of cell death after subtracting the percentage of untreated and unlabelled dead cells.

Migration in Boyden chambers

The cells were deprived of serum overnight. 50000 cells were plated in triplicate in serum-free medium into the upper compartment of pre-wetted inserts (24-well plate fit, translucent PET membrane, 8·0 μm pore size, Falcon, Thermo Fisher). Medium containing 10% serum or CXCL8 (200ng/ml, Peprotech) was added to the lower compartment. After 24 hours, migrated cells were fixed with 3% paraformaldehyde and stained with Crystal Violet. Bright field images were taken using an Evos XL Core Cell Imaging System (Thermo Fisher) with 10× objective. Cells were counted using ImageJ software (NIH).

Invasion assays

5000 cells were plated in 48 multi-well plates coated with agarose 0·8%. After two days, spheroids were mixed with 100μl of matrigel mix (200μl of matrigel (Corning Matrigel Matrix, Fontenay-sous-Bois, France), 100 μl collagen I (8 mg/ml, Corning), 80 μl HEPES 1M and 320μl respective medium) in 12 multi-well plates. 1h after seeding, 1ml of the respective medium was added with or without treatment. Images were recorded each day with the Evos XL Core Cell Imaging system (Thermo Fisher).

XTT assays

5000 Daoy/ONS-76 and 50 000 HD-MB03/D341 cells were exposed to different concentrations of C29 in 96-well plates for 48h. 50µl/well of XTT solution (Cell Proliferation kit II, Sigma-Aldrich) were added to measure cell metabolism at 490 nm with GloMax-Microplate (Promega).

Pseudo-vessels formation assays

The cells were deprived of serum overnight. They were then seeded (75000 Daoy cells, 200000 ONS -76 cells or 300000 HD -MB03 cells per well) in 24-well plates coated with Matrigel Matrix, hESC-Qualified (VWR). Plates were then incubated at 37 °C, 5% CO2 for 24 hours. Representative images were taken with the Evos XL Core Cell Imaging System at low magnification (4X). For each image, we determined the number of meshes (number of geometric units of the net formed by the cells seeded on the matrigel surface).

RT-qPCR

RNA purified with the RNeasy Mini Kit (Qiagen, Courtabœuf, France) and reverse transcription was performed using the "QuantiTect Reverse Transcription Kit" (Qiagen), according to the manufacturers' instructions. The PCR program was executed on "Professional Basic Thermocycler" (Biometra, Jena, Germany). Real-time PCR reactions were carried out in triplicates, on the StepOne Plus Real-time PCR system (Thermo Fisher), using the Takyon™ ROX SYBR 2X MasterMix dTTP Blue (Eurogentec). Specific primers were synthesized by Sigma-Aldrich or Eurogentec. The 36B4 mRNA was used as a normalization control. Data were analysed as comparative Ct (ΔΔCt; relative quantitation).

Enzyme-linked immunosorbent assays (ELISA)

Cells were seeded in 12-well plates in triplicate (50000 cells/well for Daoy/DR1/DR2 or ONS -76 cells, 100000 cells/well for HD -MB03/HR1/HR2 or 250000 cells/well for D341Med). Cells were grown for 48 hours at 37 °C in a humidified atmosphere with 5% CO_2. Supernatants were then separated and cells from each well were counted to normalize the data. Cytokines were quantified from the conditioned medium using the Peprotech (Human GRO -/MGSA CXCL1 #900-K38; Human GRO - CXCL2 #900-M120, Human NAP -2 CXCL7 #900-K40, Human IL -8 CXCL8 Kit #900-M18) or R&D (CXCL5/ENA-78 #DY254) System ELISA kit according to the manufacturer's recommendations. Data are expressed as secreted cytokines in pg/ml/10^6 cells/48 h.

Protein extraction and immunoblotting

Cells were cultured to sub confluency and lysed in 60 mM Tris-Cl pH 6·8, 2% SDS, 10% glycerol, 5% β-mercapto-ethanol, 0·01% bromophenol blue).

Lysates were sonicated for 25 seconds, and protein amount was determined by the Pierce™ BCA Protein Assay Kit assay (Thermo Fisher). Sample proteins were reduced by heating at 95 °C for 5 min. 30 µg of proteins were separated on 10% SDS-polyacrylamide gels and transferred on PVDF membranes in Tris-Glycine buffer (25 mM Tris, 192 mM glycine, pH 8·3) + 20% ethanol (v:v). Membranes were blocked with 3% BSA, at room temperature for 1 h, then immunoblotted overnight with primary antibodies diluted in 3% BSA, at 4 °C. The following primary antibodies were used: AKT (CST, #9272S), phospho-AKT (CST, #9271), ERK (CST, #4695S), phospho-ERK (CST, #4370S), HSP90 (CST, #4877S), ARD1 (homemade polyclonal rabbit antibody). Membranes were incubated with HRP-conjugated secondary antibodies anti-rabbit IgG 1/5000^e (Promega), at room temperature, for 1h30. After washing, the Advansta WesternBright Quantum HRP substrate (Diagomics, Blagnac, France) was used as detection reagent.

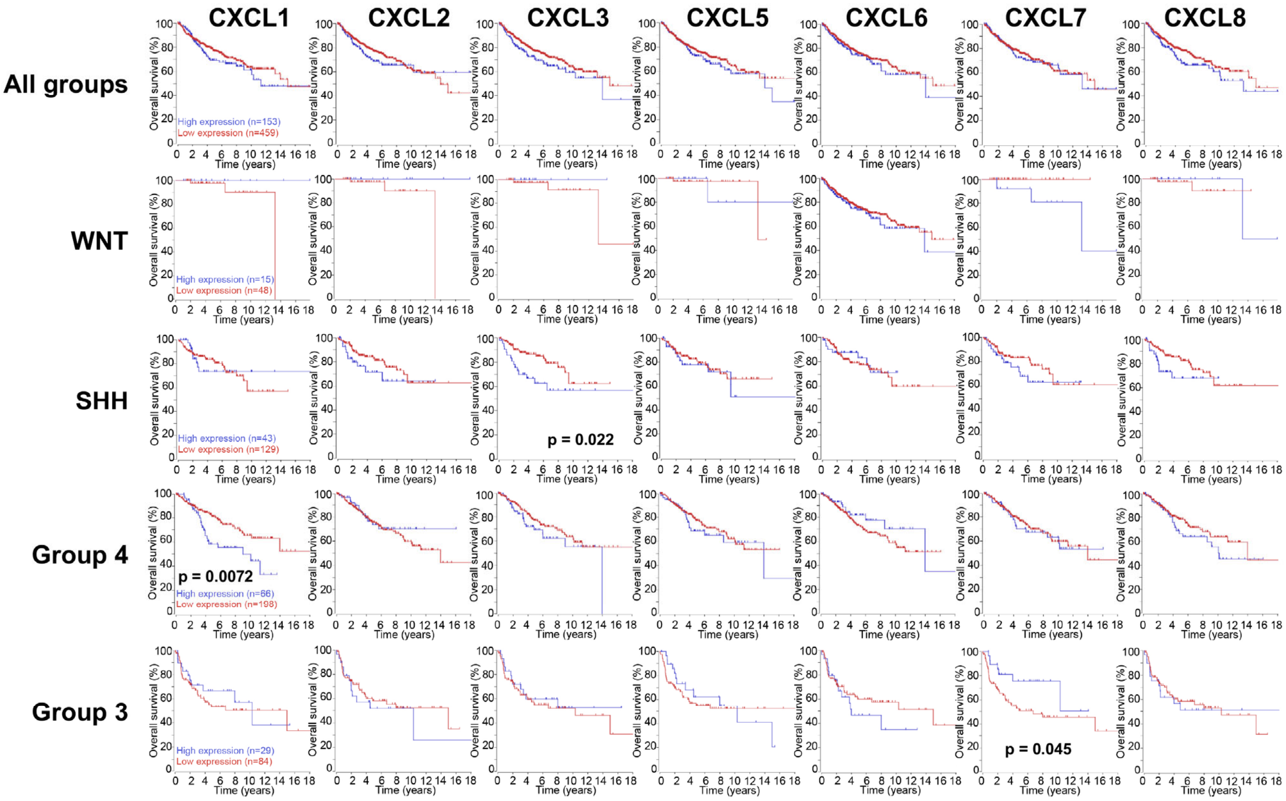

Figure S1. ELR+CXCL expression in MB patients. Analysis of overall patient survival (Kaplan-Meier curves) as a function of the rate of ELR+CXCL mRNA (CXCL1, 2, 3, 5, 6, 7, 8) in the Cavalli database of R2 software, in all groups and in the 4 subgroups: WNT, SHH, Group 4 and Group 3, cut off = last quartile. When significant p-values are indicated.

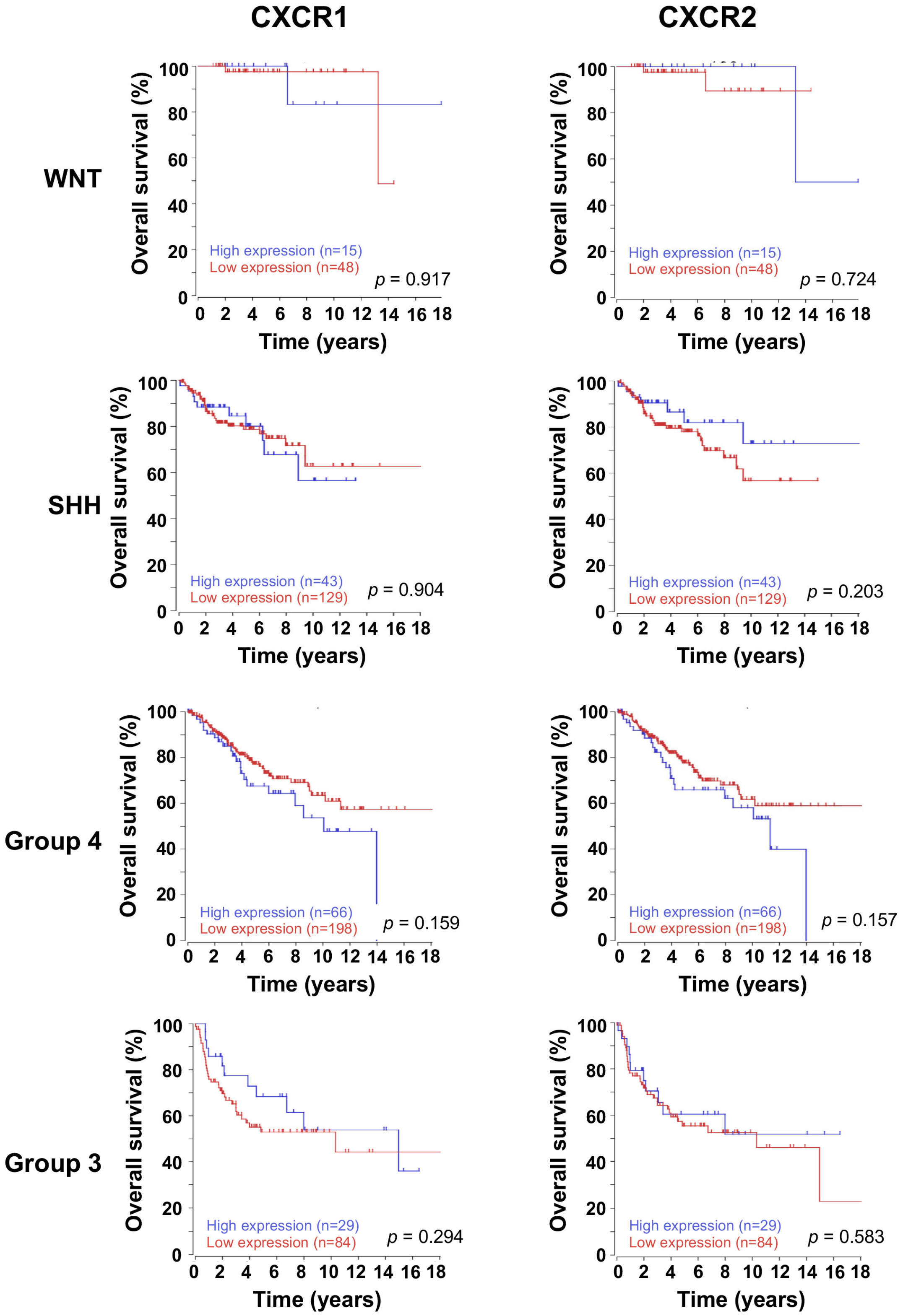

Figure S2. CXCR expression in MB patients. Analysis of overall patient survival (Kaplan-Meier curves) as a function of CXCR1 or 2 mRNA rate in the Cavalli database of the R2 platform, in WNT, SHH, Group 4 and Group 3, cut-off = last quartile. Although non-significant, p value are indicated.

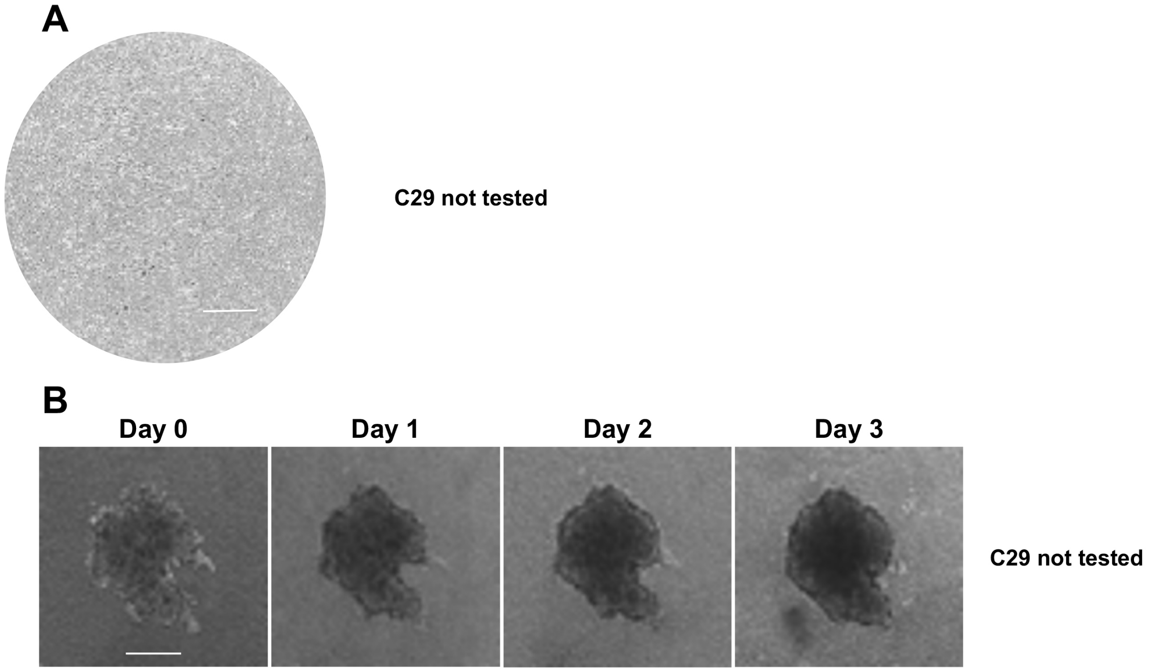

Figure S3. Migratory and invasive capacity of HD -MB03 cells. A. Migration of HD-MB03 cells through the Boyden chamber. Scale=1000μm. B. HD-MB03 spheroid invasion using 3D culture cell assays. Scale=500μm.

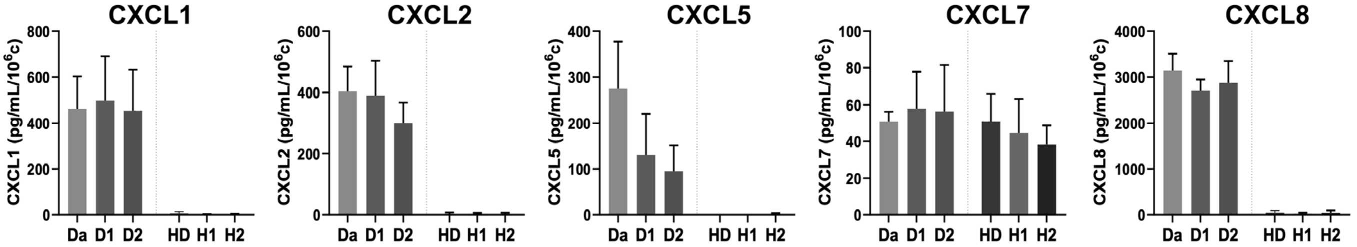

Figure S4. Radiation-resistant MB cells expressed ELR+CXCL-CXCR markers. ELR+CXCL (CXCL1, 2, 5, 7, 8) dosage by ELISA assay in naïve and resistant (R) Daoy (Da, DR1, DR2), and HD-MB03 (HD, HR1 and HR2), n=3. No statistical difference was observed between naïve and R cells. For this figure, Daoy = Da, DR1 = D1, DR2 = D2, HD-MB03 = HD, HR1 = H1 and HR2 = H2.
